# Unveiling the Role of A2AR Dysregulation in Olfactory Dysfunction and Amyloidogenesis in Alzheimer’s Disease

**DOI:** 10.1002/alz.092055

**Published:** 2025-01-03

**Authors:** AILIYA AILIYA, Ligao BAO, XIJIRI XIJIRI, Shigeru KAKUTA, Eerdunfu Eerdunfu

**Affiliations:** ^1^ The University of Tokyo, Bunkyo‐ku, Tokyo Japan

## Abstract

**Background:**

Alzheimer’s Disease (AD) manifests early in the olfactory system, yet its precise role in the pathophysiology of AD remains elusive. This study aims to elucidate the progression of olfactory dysfunction in AD by investigating the dysregulation of the adenosine 2A receptor (A2AR) and its potential involvement in the formation of abnormal plaques and tangles. A2AR plays a pivotal role in modulating synaptic transmission and neuroinflammation by regulating both neurons and glial cells. Its regulatory impact on olfactory function prompted the hypothesis that A2AR tightly binds to the Amyloid Precursor Protein (APP), influencing the formation of Amyloid β‐Protein (Aβ).

**Method:**

To explore the impact of A2AR dysregulation in the olfactory system of AD, we examined A2AR expression patterns in the olfactory bulb, striatum, and hippocampus of single amyloid precursor protein knock‐in (APP^NL‐G‐F^) mice. Additionally, in vitro experiments like RNAi knockdown using SH‐SY5Y cell line were conducted to mimic the regulatory effects of A2AR on APP and Aβ, providing insights into the pathological mechanisms.

**Result:**

Our findings revealed an age‐dependent increase in A2AR expression in the olfactory bulb, hippocampus, and striatum in APP^NL‐G‐F^ mice. Notably, A2AR expression precedes APP expression in the olfactory bulb, suggesting A2AR has a regulatory role in amyloidogenesis. Furthermore, co‐expression analysis revealed a significant association between A2AR and APP, underscoring their potential interaction in AD pathology. The APP expression pattern will be led by A2AR expression from glomerular layer to the granule cell layer of the APP^NL‐G‐F^ mice olfactory bulb. Additionally, modulating A2AR expression alters cellular responses to caffeine and adenosine on SH‐SY5Y cell line and regulating APP expression, implicating its involvement in synaptic transmission and amyloid pathology.

**Conclusion:**

This study underscores the pivotal role of A2AR dysregulation in olfactory dysfunction associated with AD. The observed tight binding of A2AR with APP implies its direct involvement in Aβ formation. These findings highlight the significance of A2AR dysregulation in olfactory dysfunction and amyloidogenesis, suggesting novel therapeutic targets and early diagnosis for AD intervention.

